# Maternal pre-pregnancy anemia and childhood anemia in Indonesia: a risk assessment using a population-based prospective longitudinal study

**DOI:** 10.4178/epih.e2022100

**Published:** 2022-11-01

**Authors:** Fadila Wirawan, Dieta Nurrika

**Affiliations:** 1 Department of Public Health Nutrition, Faculty of Public Health, Universitas Indonesia, Depok, Indonesia; 2 Department of Public Health, Banten School of Health Science, South Tangerang, Indonesia

**Keywords:** Anemia, Child, Iron deficiency, Maternal health, Preconception care, Pregnancy

## Abstract

**OBJECTIVES:**

Anemia in children under 5 years of age is often overlooked despite its detrimental effects. The public health approach to anemia prevention includes the maternal pre-pregnancy phase. This study investigated the association between pre-pregnancy anemia and the risk of anemia in children under 5 years of age.

**METHODS:**

This cohort study included non-pregnant women from the 2007 Indonesian Family Life Survey (IFLS) and their children under 5 in the 2014 IFLS. The anemia status of mothers and children was determined based on hemoglobin (Hb) levels using Hemocue. Mantel-Haenszel adjusted relative risks (aRRs), including risk stratification by covariates, were used for the final risk assessment.

**RESULTS:**

In total, 637 children in the 2014 IFLS were included. The risk of having a child with anemia was 1.71-fold higher in women with pre-pregnancy anemia than in women without pre-pregnancy anemia (aRR, 1.71; 95% confidence interval [CI], 1.03 to 2.85). After risk stratification based on potential confounding variables, maternal pre-pregnancy anemia remained an independent risk factor for anemia in children who still breastfed at the time of data collection (relative risk [RR], 2.11; 95% CI, 1.16 to 3.86), in children who were given water earlier than 6 months of age (RR, 2.08; 95% CI, 1.20 to 3.61), in children of mothers with a normal or underweight pre-pregnancy body mass index (RR, 1.94; 95% CI, 1.20 to 3.14), and in children of mothers without current anemia (RR, 2.20; 95% CI, 1.21 to 3.99).

**CONCLUSIONS:**

Pre-pregnancy anemia increased the risk of childhood anemia. A public health approach emphasizing pre-conception maternal health would enable better maternal and child morbidity risk prevention.

## INTRODUCTION

Anemia in women and children poses a global public health problem that remains unresolved. The effects of anemia in women and children are not only detrimental to health, but also negatively affect the quality of human resources in the future [1,2]. Anemia in women of childbearing age and in children younger than 5 years old is common in many lower-middle-income countries [3]. In Asia, anemia remains one of the most prevalent severe maternal morbidities [4].

In response to the high prevalence of maternal anemia, multiple countries, including Indonesia, have implemented interventions and preventive policies by providing iron supplements containing at least 60 mg of elemental iron to adolescent girls [5,6]. However, childhood anemia remains overlooked despite the substantial effects of anemia in children under 5 years old on their development, cognition, growth, immunity, and future academic performance [7,8]. According to the Indonesian Basic Health Research report on childhood anemia in 2013, Indonesia had a 28.1% prevalence rate of childhood anemia [9], and it rose to 38.5% in 2018 [10]. The prevalence rate in Indonesia was higher than that of the surrounding countries in Southeast Asia [11].

Nutrition, maternal health, childhood illness, household economic level, and the mother’s education level have been reported as determinants of childhood anemia [12]. Iron deficiency anemia (IDA) is the most common type of anemia in women and children, which is caused by micronutrient deficiency [8]. IDA is estimated to affect 63% of adolescent girls in Indonesia [13]. Anemia that occurs before pregnancy and is left untreated can increase the metabolic demand during pregnancy. Pregnant women require significantly more iron than non-pregnant women. Approximately one-third of the need for iron during pregnancy is associated with fetal and placental demand. Therefore, an unmet iron demand, especially if anemia was already present, could impact maternal and fetal health [14].

Pre-pregnancy intervention or iron supplementation pre-conception corresponds to increases in iron storage in infants [15], suggesting that pre-pregnancy anemia may also contribute to anemia in children. Multiple studies have also observed association between maternal anemia during pregnancy and the child’s health outcomes. In addition, maternal anemia has a reported association with anemia in children under 5 [8,16]. Anemia during the early gestational period can also influence birth outcomes [17]. However, little is known about pre-pregnancy or pre-conception anemia’s influence on child anemia, especially in children under 5 years old.

Interventions to prevent childhood morbidity, including anemia, have also suggested moving toward pre-conception care [18]. Moreover, pregnancy is not always a planned event. Addressing maternal health concerns in the pre-pregnancy phase could be an effective strategy for improving children’s health outcomes. Using prospective cohort data, the relative risk (RR) of childhood anemia can be estimated to bridge the existing knowledge gap. The current study aimed to analyze the association between pre-pregnancy anemia and the risk of anemia in children under 5 years old in Indonesia using population-based cohort data. The results of this study could identify several variables associated with childhood anemia and increase the understanding of pre-pregnancy anemia and childhood anemia in Indonesia.

## MATERIALS AND METHODS

### Study population and setting

This prospective cohort study used data from the Indonesian Family Life Survey (IFLS) from 2007 to 2014. The IFLS is an ongoing longitudinal socioeconomic and health survey that was initiated in 1993. The IFLS sample population comprised individuals from 13 provinces, covering 83% of the Indonesian population. Enumeration areas were randomly chosen within the provinces based on the SUSENAS, a national socioeconomic survey, to create the data sample frame. The households to be surveyed were randomly selected based on regional government statistics listings [19,20].

**Figure 1:**
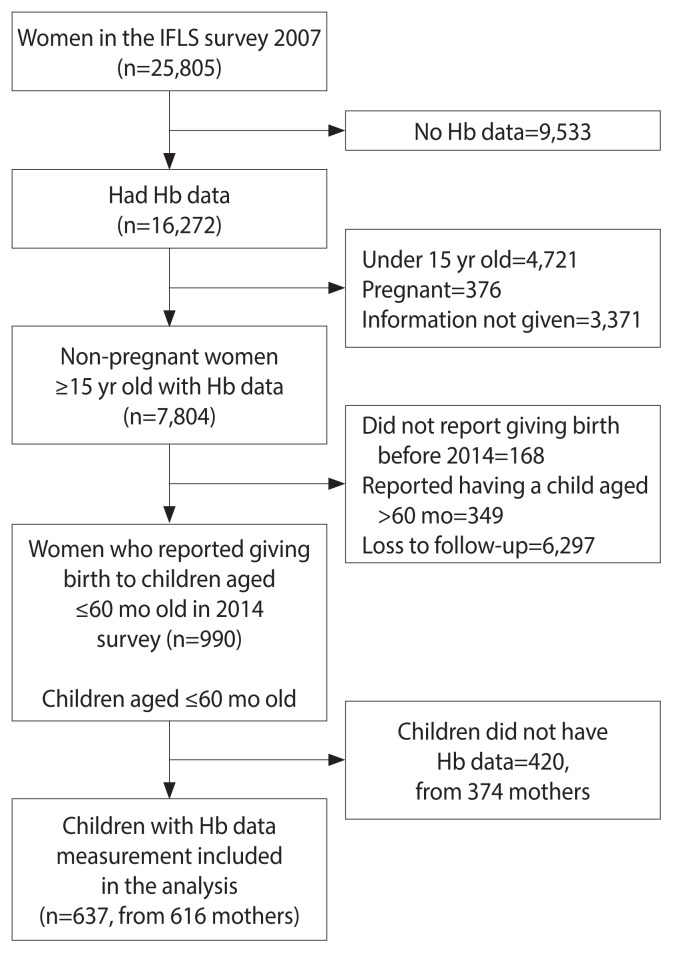
Study subject selection scheme. IFLS, Indonesian Family Life Survey; Hb, hemoglobin.

Pre-pregnancy was defined as the period before pregnancy occurs, either before the first pregnancy or between pregnancies [21]. This study observed the status of anemia in non-pregnant women and the occurrence of anemia in their children under 5 years old as the outcome. The inclusion criteria were women of reproductive age (15 years and above) who participated in the 2007 IFLS survey, were not pregnant, had data for their measured hemoglobin (Hb) status, and had given birth to a child aged 60 months or under in the 2014 survey. Children without Hb data were excluded from the analysis. A total of 25,805 women joined the survey in 2007, but only 7,804 were not pregnant, aged 15 years or older, and had Hb data. We identified follow-up data from the 2014 survey on 990 mothers and 1,057 of their children aged 60 months and under. Some of the children were excluded from this study due to missing Hb data, leaving 637 children under 5 years old from 616 mothers to be included in the analysis (Figure 1). To address possible selection bias, a comparative analysis was conducted to compare the baseline characteristics of the included and excluded mothers with children under 5 (Supplementary Material 1). The distribution of pre-pregnancy anemia, pre-pregnancy body mass index (BMI), iron supplement consumption, number of iron supplements consumed in the previous 4 weeks, smoking status, area of residence, and province of residence were similar (p≥0.05), while the distribution of age and education differed (p<0.05) between the included and excluded groups.

A minimum sample of 244 women was required based on a sample size calculation and a 19.7% prevalence rate of anemia in adult women identified in a national report conducted at the time of data collection [22]. Based on the 95% confidence level and sample size of 637, the margin of error of this study was 3.09%.

### Anemia

The anemia status of mothers during pre-pregnancy was considered the exposure. The primary outcome variable was the anemia status of their children under 5 years old. We also analyzed the anemia status of the mothers and the anthropometry status of the children as the secondary outcome at the endpoint in 2014. In the 2007 and 2014 IFLS surveys, the Hb, weight, and height of the participants were measured by trained health workers. Hb was measured in-person using a point-of-care Hb test device (Hemocue). Anemia in non-pregnant women was defined as an Hb measurement below 12 g/dL, while anemia in children was defined as an Hb measurement below 11 g/dL, according to WHO criteria. In this study, anemia status was classified as either “anemia” (Hb <11 g/dL in children or Hb <12 g/dL for women) or “no anemia” (Hb ≥11 g/dL in children and Hb ≥12 g/dL in women).

### Possible confounding variables

Possible confounding variables were classified as maternal-related, child-related, and socio-demographic variables. Maternal-related variables included the mother’s age when pregnant (grouped into quartiles), pre-pregnancy anemia status in 2007 (anemia or no anemia), anemia status in 2014 (anemia or no anemia), iron supplementation status during pre-pregnancy (yes or no), number of iron supplement tablets consumed in the previous 4 weeks in 2007 (grouped into quartiles), pre-pregnancy BMI, and smoking history during pre-pregnancy (yes or no). Child-related variables included gender (girl or boy), birth weight (<2,500 or ≥2,500 g), breastfeeding history (yes or no), breastfeeding status at the endpoint (yes or no), whether the child was given water before 6 months of age (yes or no), whether the child was given food before 6 months of age (yes or no), the age at which the child was weaned from breastfeeding (before 2 or after 2 years), whether the child consumed beef in the previous week (yes or no), whether the child was given vitamin A in the previous 6 months (yes or no), and reported symptoms of illness (yes or no). Socio-demographic variables included the area of residence (urban and rural), region (Sumatera, Java and Bali, or other provinces), household economic level (grouped into quartiles based on per capita expenditures), mother’s education level, house latrine condition, and drinking water source.

Maternal pre-pregnancy BMI was calculated based on the weight and height measured during pre-pregnancy. BMI was classified based on WHO criteria: 18.5 kg/m^2^ was considered underweight, 18.5 kg/m^2^ to 24.9 kg/m^2^ was considered normal; 25.0 kg/m^2^ to 29.9 kg/m^2^ was considered overweight, and 30.0 kg/m^2^ or above was considered obese. The children’s weight was categorized as “underweight” if their z-score for weight-for-age was ≤-2 standard deviations (SDs); otherwise, they were categorized as “not underweight.” Children were classified as “stunted” when their height-for-age z score was ≤-2 SDs; otherwise, they were classified as “not stunted.”

Other variable data were obtained through interviews using the IFLS questionnaire, which is described in previous studies [19,20]. Iron supplementation status, birth weight, breastfeeding history, the number of supplements taken, and the children’s symptoms of illness were self-reported by the mothers. Information on iron supplementation history and the number of tablets consumed were limited to the previous 4 weeks. Birth weight was self-reported by the mothers and grouped based on the WHO classification for low birth weight of below 2,500 g [23]. Whether the children were fed water and food early were defined based on WHO criteria, which specifies a cut-off of 6 months old for exclusive breastfeeding [24]. Children’s symptoms of illness were based on whether the children had any symptoms of a possible infection or chronic disease or experienced blood loss, fever, diarrhea in any form (bloody, mucous, or liquid), or a bloody cough in the previous 4 weeks as reported by their mothers. The mothers’ education levels were classified as “no education,” “elementary school or equal,” “middle school or equal,” “high school or equal,” “undergraduate or graduate,” or “post-graduate.” House latrine condition and drinking water source variables were proxies for the contributing risk of parasite infection related to anemia [25]. House latrine condition was categorized as “using own toilet,” “communal toilet,” “public toilet,” “creek or river,” “yard,” “sewer,” “pond,” and “sea.” The drinking water source was classified as “pipe water,” “well water,” “spring water,” “rain collection,” “river,” “water collection,” and “bottled water.”

### Statistical analysis

Data were extracted from the 2007 and 2014 IFLS datasets. The IFLS contains unique personal identification numbers that enabled data on individuals to be tracked from 2007 to 2014. After screening the data according to the inclusion and exclusion criteria, the data were checked for duplicate entries and extreme outliers and entered into statistical software. Covariate data for every variable were not available for all participants; therefore, the covariate number may not have matched the total number of subjects, and all missing values were excluded from analysis.

SPSS version 26 (IBM Corp., Armonk, NY, USA) and StataBE 17 (StataCorp., College Station, TX, USA) were used for the analysis. The Kolmogorov-Smirnov test was conducted to confirm the normality of data distribution. Values for the categorical variables were presented as numbers and percentages. Values for numerical variables were presented in terms of the mean and SD since the Hb levels of children and mothers during pre-pregnancy had a normal distribution. The chi-square test was used to identify associations between maternal pre-pregnancy anemia as the exposure and the possible confounding variables, as well as the children’s anemia status and the possible confounding variables. A p-value of <0.1 was considered to show a non-significant correlation, while a p-value of <0.05 with a corresponding 95% confidence interval (CI) was considered to indicate statistical significance; variables that showed statistically significant relationships according to this definition were included in the adjusted model as potential confounding variables. A RR assessment was conducted to calculate the risk of maternal pre-pregnancy anemia exposure for each outcome. The adjusted RR was obtained using Mantel-Haenszel analysis. A risk stratification analysis was conducted to evaluate the stratified RR of pre-pregnancy anemia according to the potential confounding variables related to childhood anemia.

### Ethics statement

This study data were publicly accessible with the permission of RAND Corporation, which is the institute that conducts the IFLS survey and owns the data. The IFLS survey method was reviewed and approved by the Universitas Gadjah Mada Institutional Review Board with the ethical clearance No. s0064-06-01-CR01.

## RESULTS

This study included 616 mothers with 637 children under 5 years old (mean age: 41.04±13.34 months). The mothers’ ages ranged from 15 years to 44 years in 2007 (mean age: 27.10±5.30 years). The mean age at pregnancy was 30.6±5.30 years. The mean time until pregnancy was 43.00±13.30 months after baseline data collection. The prevalence of anemia in mothers in 2007 was 21.30% (mean Hb: 12.83±1.28 g/dL) and increased to 31.30% in 2014 (mean Hb: 12.44±1.51 g/dL). The prevalence of anemia in children under 5 years old was 11.50% (mean Hb 11.01±1.34 g/dL). The incidence of having a child under 5 years old with anemia was 18.12% among pre-pregnancy mothers.

The distributions of variables based on maternal pre-pregnancy anemia status are shown in Table 1. The baseline parameters were age, iron supplement consumption, the number of iron supplements consumed in the previous 4 weeks, pre-pregnancy BMI, smoking status, area of residence, region, and education level. In 2007, 86.30% of mothers consumed iron supplements in the previous 4 weeks (mean: 110.87±122.80 tablets). The area of residence tended to correlate with pre-pregnancy anemia status.

Table 2 shows the risk factors for childhood anemia. A total of 18.12% of mothers with pre-pregnancy anemia had children with childhood anemia, while 9.92% of mothers without pre-pregnancy anemia had children with childhood anemia. Among mothers with anemia at the endpoint, 14.57% had children with childhood anemia, while 10.07% of mothers without anemia at the endpoint had children with childhood anemia. Maternal pre-pregnancy anemia status was significantly correlated (p=0.01) with childhood anemia. Other variables also showed significant correlations, including maternal pre-pregnancy BMI (p=0.03), stunting (p< 0.01), continued breastfeeding at the endpoint of data collection (p<0.01), and being fed water before the age of 6 months (p=0.01). The mother’s anemia status in 2014, birth weight, underweight status, and the child having symptoms of illness showed non-significant correlations (p=0.09, 0.07, 0.06, and 0.09, respectively).

Maternal and child outcomes according to the presence of maternal pre-pregnancy anemia are shown in Table 3. Child anemia was the primary outcome, while the child’s birth weight, stunting status, and underweight status, as well as maternal anemia status in 2014, were analyzed as secondary outcomes. Maternal pre-pregnancy anemia increased the risk of child anemia by 1.88 times compared to mothers without pre-pregnancy anemia as a reference (RR, 1.88; 95% CI, 1.21 to 2.94). The risk remained significantly high after adjusting for maternal pre-pregnancy BMI, child stunting status, continued breastfeeding, and if the child was fed water before 6 months of age (aRR, 1.71; 95% CI, 1.03 to 2.85). Among the secondary outcomes, child stunting tended to be affected by maternal pre-pregnancy anemia (RR, 1.15; 95% CI, 1.00 to 1.32). Maternal pre-pregnancy anemia also significantly increased the risk of anemia in the mother later in life (RR, 1.87; 95% CI, 1.50 to 2.34). The risk remained significantly increased after adjustment for the mother’s pre-pregnancy BMI and area of residence (aRR, 1.77; 95% CI, 1.28 to 2.45).

Table 4 shows further risk stratification of maternal pre-pregnancy anemia based on potential confounding variables related to child anemia. Maternal pre-pregnancy anemia remained an independent risk factor for childhood anemia in children who were still being breastfed (RR, 2.11; 95% CI, 1.16 to 3.86), children who were given water before 6 months of age (RR, 2.08; 95% CI, 1.20 to 3.61), children with mothers with a normal or underweight pre-pregnancy BMI (RR, 1.94; 95% CI, 1.20 to 3.14), and children of mothers without anemia in 2014 (RR, 2.20; 95% CI, 1.21 to 3.99). Stunted children of mothers with pre-pregnancy anemia had a higher risk of child anemia; however, the effect was not statistically significant (RR, 1.64; 95% CI, 1.00 to 2.70).

## DISCUSSION

This study analyzed the status of anemia in 616 non-pregnant women in 2007 as an indicator of childhood anemia in 637 children in 2014. The current reported anemia prevalence rate in children under 5 years old was lower than the previously reported national prevalence rate [11]. However, the maternal anemia rate in 2007 and 2014 was higher in this study than the national prevalence rates of 19.7% in 2007 and 23.9% in 2013 [9,22]. Maternal pre-pregnancy anemia, maternal pre-pregnancy BMI, child stunting, continued breastfeeding, and whether the child was given water before 6 months of age were risk factors associated with childhood anemia. Maternal pre-pregnancy anemia increased the risk of childhood anemia by 1.71 times after adjusting for potential confounding variables. The increased risk of the childhood anemia in the children of women with pre-pregnancy anemia was independently significant, even increased, when the child was still breastfed, given water before 6 months of age, the mother had a normal or underweight pre-pregnancy BMI, and regardless the mother’s current no anemia status. Stunting was an independent confounding variable related to childhood anemia.

The current results suggest that maternal pre-pregnancy anemia increased the risk of anemia in children under 5. While pre-pregnancy or preconception anemia has been examined in multiple previous studies, its effect on childhood anemia has not yet been determined [15,26]. An earlier study of Chinese women found that moderate pre-pregnancy anemia was associated with increased low birth weight and fetal growth restriction [27]. In another study examining iron supplementation, preconception iron supplementation increased maternal ferritin during pregnancy and postpartum as well as increased iron stores in infants. However, the previous studies did not analyze the association between maternal pre-pregnancy anemia status and infant anemia [15]. This study not only identified an increased risk of child anemia associated with pre-pregnancy anemia, but also a correlation between current maternal anemia and child anemia. The relationship could be mediated by pre-pregnancy anemia status since the current study observed that mothers with pre-pregnancy anemia also had an increased risk of anemia after pregnancy. In addition, the risk of child anemia following pre-pregnancy anemia was consistent in children with mothers who were not currently anemic.

Maternal iron deficiency during pregnancy is already known to be one of the leading causes of anemia in mothers, infants, and young children [8,28]. Iron is important for Hb production. Iron intake, either dietary or supplemental, builds the body’s iron stores that help prevent anemia [29]. Besides iron, vitamin A is also involved in red blood cell growth and reduces anemia caused by infection [30]. Iron deficiency during pregnancy can cause low iron stores in the newborn and can contribute further to the development of childhood anemia [28]. A cohort study conducted in China also found that maternal anemia increased the risk of childhood anemia at 6 months [31]. Iron supplementation in women and vitamin A supplementation in children have already been incorporated in public health policy to prevent anemia in Indonesia. However, based on the current study, the prevalence of anemia in both women and children remains high despite iron intake in mothers and vitamin A intake in children.

Children’s nutritional status and anemia were correlated in the current study, which corresponds to the findings related to another population-based cohort [32]. Although an underweight BMI classification only showed a non-significant correlation, stunting was a confounding variable in child anemia independent of maternal pre-pregnancy anemia. Simultaneous anemia and stunting have been reported in many other studies, including in studies of Indonesian populations [7,33,34]. This association may be due to overlapping risk factors [28,35]. Anemia strongly correlates with growth failure in children, and underweight and stunting can be caused by malnutrition and a likely micronutrient deficiency, as well as possible impaired iron metabolism and hematopoiesis, all of which contribute to anemia [7,36].

Maternal pre-pregnancy BMI is also correlated with childhood anemia. Pre-pregnant mothers with anemia with a BMI classification of normal or obese have an increased risk of giving birth to a child with anemia. Previous studies have found that overweight women had an increased probability of high dietary iron, which could have a protective effect on child anemia. As mentioned, mothers pass their iron stores to their offspring [28]. However, previous studies have reported that maternal obesity rather than underweight was correlated with child anemia [31]. The current study observed a correlation between pre-pregnancy BMI and childhood anemia. After stratifying the risk of pre-pregnancy anemia based on BMI, the risk was significant in mothers with a pre-pregnancy BMI classification of underweight or normal but not in mothers with a BMI classification of overweight or obese. Further assessment is required to properly interpret and understand the relationship between maternal pre-pregnancy BMI and childhood anemia.

Breast milk carries the best nutrition for infants, and breastfeeding may have a protective effect against anemia in infants, especially in the first 6 months, according to multiple previous studies [37,38]. However, if a mother is iron deficient, the iron levels in breast milk may drop as breastfeeding progresses [28]. The population in the current study had a high prevalence of anemia, particularly among mothers in 2014. Maternal pre-pregnancy anemia also increased the risk of maternal anemia in the population at the data collection endpoint. Moreover, the presence of maternal pre-pregnancy anemia in mothers who were still breastfeeding a child showed a more than 2-fold increased risk of having an anemic child compared to mothers without pre-pregnancy anemia. The effect of pre-pregnancy anemia on anemia in children also did not continue in children who were no longer breastfed, suggesting the possibility that mothers with pre-pregnancy anemia may not pass on sufficient iron in their breast milk, leading to IDA. Children who were given water before 6 months of age and who were not exclusively breastfed also had an increased risk of anemia when their mothers had pre-pregnancy anemia. The increased risk is likely due to the inadequate passing of iron stores [28]. However, further information on the daily feeding habits of infants, including the quantity and type of water they were given, is needed to draw a conclusion.

Pre-pregnancy or preconception care is a recently introduced concept in health risk prevention to improve maternal health and gestational outcomes [26]. Shifting the disease prevention target to pre-pregnancy could contribute to earlier maternal and child risk assessment and provide a better chance of avoiding morbidity [21]. This study was the first known longitudinal study to analyze the association between pre-pregnancy anemia and the risk of anemia in children under 5 years old among the Indonesian population. While many observations have been made related to exposure to anemia during pregnancy and the child’s outcomes, few studies have examined the pre-pregnancy phase in relation to the child’s health. This study examined the risk of childhood anemia related to exposure before pregnancy and observed the child’s outcomes. The analysis also assessed variables that were previously identified as determining factors for child anemia. Another strength of this study is the national representativeness of the data since the IFLS study population was taken from randomized participants across Indonesia. Although there was a relatively high loss to follow-up and a high number of excluded participants, the comparison of baseline characteristics between the included and excluded groups were mostly similar, suggesting that the participants in this study are representative of mothers with children under the age of 5 years overall. Data were also collected using calibrated tools to measure Hb and anthropometry. Therefore, the findings of this study could provide new insights into population-wide health approaches to reduce child anemia and promote pre-pregnancy anemia prevention in women of childbearing age.

Some limitations, however, could not be avoided. The analysis was based on secondary data from a population-based survey, and some factors may not have been accounted for. In addition, some variables were self-reported and could be biased as a result. The loss to follow-up was also relatively high. Some data were effectively lost to follow-up due to a mismatch between the person’s identification code at the baseline and endpoint. The distribution of age and the mother’s education level differed between the included and excluded population, which may have contributed to selection bias. The child illness data were also limited, and information about some illnesses that may cause anemia was missing, such as information about infections, chronic diseases, and genetic diseases. The IFLS survey also did not include a thorough dietary intake assessment, and other high-iron foods and iron supplements in children were not included in the analysis. Moreover, the population of this study was limited to the Indonesian population, and the results may not be generalizable to other populations. An additional primary cohort survey that controls for confounding variables or a randomized controlled trial for the development of pre-pregnancy anemia intervention is needed to draw more detailed and informed conclusions.

In conclusion, pre-pregnancy anemia contributes to the risk of anemia in children under 5 years of age. Although other confounding variables also play some role in the development of childhood anemia, pre-pregnancy health in mothers contributes to the health of their offspring. The strength of the evidence regarding the association between pre-pregnancy anemia and childhood anemia supports the belief that maternal health initiatives should look beyond the 1,000 days of life.

## Figures and Tables

**Table 1:** Distribution of variables based on maternal pre-pregnancy anemia status (n=637)

Variables	n	Maternal pre-pregnancy anemia status		p-value^1^
		Anemia	No anemia	
Baseline parameters in 2007				
Age in 2007 (n=637)				0.19
Quartile 1 (≤23)	167	43 (25.75)	124 (74.25)	
Quartile 2 (24–27)	181	39 (21.55)	142 (78.45)	
Quartile 3 (28–30)	135	21 (15.56)	114 (84.44)	
Quartile 4 (>30)	154	35 (22.73)	119 (77.27)	
Pre-pregnancy iron supplement (n=614)				0.45
Yes	530	61 (11.51)	469 (88.49)	
No	84	6 (7.14)	78 (92.86)	
No. of iron supplement tablets consumed in the previous 4 wk (2007) (n=467)^2^				
Quartile 1 (≤21)	124	33 (26.61)	91 (73.39)	0.28
Quartile 2 (21–80)	116	19 (16.38)	97 (83.62)	
Quartile 3 (81–180)	112	26 (23.21)	86 (76.79)	
Quartile 4 (>180)	115	24 (20.87)	91 (79.13)	
Pre-pregnancy body mass index (n=631)				0.83
Underweight	64	14 (21.88)	50 (78.12)	
Normal	405	88 (21.73)	317 (78.27)	
Overweight	135	31 (22.96)	104 (77.04)	
Obese	27	4 (14.81)	23 (85.19)	
Smoking during pre-pregnancy (n=635)				0.61
Yes	4	1 (25.00)	3 (75.00)	
No	631	72 (11.41)	559 (88.59)	
Area of residence in 2007 (n=358)				0.06
Urban	182	53 (29.12)	129 (70.88)	
Rural	176	36 (20.45)	140 (79.55)	
Region in Indonesia (n=527)				0.89
Sumatera	126	26 (20.63)	100 (79.37)	
Java and Bali	280	60 (21.43)	220 (78.57)	
Other provinces	121	28 (23.14)	93 (76.86)	
Mother’s education level in 2007 (n=627)				0.15
Elementary school or equal	207	45 (21.74)	162 (78.26)	
Middle school or equal	167	34 (20.36)	133 (79.64)	
High school or equal	184	48 (26.09)	136 (73.91)	
Undergraduate or graduate	69	9 (13.04)	60 (86.96)	

Values are presented as number (%).

1 Chi-square test.

2 Based on quartiles.

**Table 2:** Risk factors for the study outcome

Variables	n	Children’s anemia status		p-value^1^
		Anemia	No anemia	
Baseline parameters in 2007				
Maternal pre-pregnancy anemia status (n=637)				0.01
Anemia	138	25 (18.12)	113 (81.88)	
No anemia	499	48 (9.62)	451 (90.38)	
Maternal pre-pregnancy iron supplement status (n=614)				0.23
Yes	530	61 (11.51)	469 (88.49)	
No	84	6 (7.14)	78 (92.86)	
No. of iron supplement tablets consumed by mother in the previous 4 wk (n=467)^2^				0.92
Quartile 1 (≤21)	124	16 (12.90)	108 (87.10)	
Quartile 2 (22–80)	116	13 (11.21)	103 (88.79)	
Quartile 3 (81–180)	112	16 (14.29)	96 (85.71)	
Quartile 4 (>180)	115	15 (13.04)	100 (86.96)	
Maternal pre-pregnancy body mass index (n=631)				0.03
Underweight	64	3 (4.69)	61 (95.31)	
Normal	405	57 (14.07)	348 (85.93)	
Overweight	135	7 (5.19)	128 (94.81)	
Obese	27	4 (14.81)	23 (85.19)	
Smoking during pre-pregnancy (n=636)				0.54
Yes	5	1 (20.00)	4 (80.00)	
No	631	72 (14.81)	559 (88.59)	
Mother’s education level in 2007 (n=631)				0.14
No education	4	2 (50.00)	2 (50.00)	
Elementary school or equal	207	27 (13.04)	180 (86.96)	
Middle school or equal	167	21 (12.57)	146 (87.43)	
High school or equal	184	16 (8.70)	168 (91.30)	
Undergraduate or graduate	69	7 (10.14)	62 (89.86)	
Endpoint parameters in 2014				
Gender, child (n=637)				0.72
Girl	283	34 (12.01)	249 (87.99)	
Boy	354	39 (11.02)	315 (88.98)	
Birth weight (n=608)				0.07
<2,500 g	32	7 (21.88)	25 (78.12)	
≥2,500 g	576	64 (11.11)	512 (88.89)	
Stunting (n=630)				<0.01
Yes	382	56 (14.66)	326 (85.34)	
No	248	14 (5.65)	234 (94.35)	
Underweight (n=637)				0.06
Yes	225	33 (14.67)	192 (85.33)	
No	412	40 (9.71)	372 (90.29)	
Breastfeeding status (n=633)				0.89
Yes	614	71 (11.56)	543 (88.44)	
No	19	2 (10.53)	17 (89.47)	
Still breastfed at endpoint (n=613)				<0.01
Yes	139	31 (22.30)	108 (77.70)	
No	474	39 (8.23)	435 (91.77)	
Given water before 6 mo of age (n=614)				0.01
Yes	485	48 (9.90)	437 (90.10)	
No	129	23 (17.82)	106 (82.17)	
Given food before 6 mo of age (n=610)				0.37
Yes	221	22 (9.95)	199 (90.05)	
No	389	48 (12.34)	341 (87.66)	
Weaned before 2 yr old (n=613)				0.54
Yes	275	29 (10.55)	246 (89.45)	
No	338	41 (12.13)	297 (87.87)	
Consumed beef in the previous wk (n=632)				0.45
Yes	347	36 (10.37)	311 (89.63)	
No	285	35 (12.28)	250 (87.72)	
Given vitamin A in the previous 6 mo (n=637)				0.50
Yes	424	46 (10.85)	378 (89.15)	
No	213	27 (12.68)	186 (87.32)	
Symptoms of illness (n=637)^3^				0.09
Yes	351	47 (13.39)	304 (86.61)	
No	286	26 (9.09)	260 (90.91)	
Maternal age when pregnant (n=637)				0.26
Quartile 1 (≤27)	198	30 (15.15)	168 (84.85)	
Quartile 2 (28–30)	141	14 (9.93)	127 (90.07)	
Quartile 3 (31–34)	142	15 (10.56)	127 (89.44)	
Quartile 4 (>34)	156	14 (8.97)	142 (91.03)	
Mother's anemia status in 2014 (n=636)				0.09
Anemia	199	29 (14.57)	170 (85.43)	
No anemia	437	44 (10.07)	393 (89.93)	
Mother’s education level in 2014 (n=634)				0.54
No education	4	1 (25.00)	3 (75.00)	
Elementary school or equal	195	23 (11.79)	172 (88.21)	
Middle school or equal	171	25 (14.62)	146 (85.38)	
High school or equal	190	17 (3.68)	173 (96.32)	
Undergraduate or graduate	72	7 (9.72)	65 (90.28)	
Post-graduate	2	0 (0.00)	2 (100)	
Latrine condition (n=633)				0.38
Own toilet	528	54 (10.23)	474 (89.77)	
Communal toilet	35	8 (22.86)	27 (77.14)	
Public toilet	18	3 (16.67)	15 (83.33)	
Creek/river	32	4 (12.50)	28 (87.50)	
Yard	11	2 (18.19)	9 (81.81)	
Sewer	2	0 (0.00)	2 (100)	
Pond	3	0 (0.00)	3 (100)	
Sea	4	0 (0.00)	4 (100)	
Drinking water source (n=617)				0.33
Pipe water	138	12 (8.70)	126 (91.30)	
Well water	243	25 (10.29)	218 (89.71)	
Spring water	48	5 (10.42)	43 (89.58)	
Rain collection	1	0 (0.00)	1 (100)	
River	6	2 (33.33)	4 (66.67)	
Water collection	4	1 (25.00)	3 (75.00)	
Bottled water	177	26 (14.69)	151 (85.31)	
Area of residence in 2014 (n=637)				0.67
Urban	364	40 (10.99)	324 (89.01)	
Rural	273	33 (12.09)	240 (87.91)	
Household economic level (n=607)^4^				0.17
Quartile 1	170	19 (11.18)	151 (88.82)	
Quartile 2	145	24 (16.55)	121 (83.45)	
Quartile 3	163	15 (9.20)	148 (90.80)	
Quartile 4	129	12 (9.30)	117 (90.70)	

Values are presented as number (%).

1 Chi-square test.

2 Based on quartiles.

3 Fever, diarrhea (any kind), or bloody cough.

4 Based on per capita expenditures.

**Table 3:** Maternal and child outcomes based on maternal pre-pregnancy anemia

Maternal pre-pregnancy anemia	Total	Outcome variables		
		n	RR (95% CI)	aRR (95% CI)
Child anemia (n=73)				
Anemia	138	25	1.88 (1.21, 2.94)^*^	1.71 (1.03, 2.85)^1,*^
No anemia	499	48	1.00 (reference)	1.00 (reference)
Child low birth weight (<2,500 g) (n=32)				
Anemia	128	5	0.69 (0.27, 1.77)	-
No anemia	480	27	1.00 (reference)	-
Child stunting (n=382)				
Anemia	135	91	1.15 (1.00, 1.32)^†^	-
No anemia	495	291	1.00 (reference)	-
Child underweight (n=225)				
Anemia	135	50	1.03 (0.80, 1.33)	-
No anemia	499	175	1.00 (reference)	-
Mother’s anemia status in 2014 (n=636)				
Anemia	138	68	1.87 (1.50, 2.34)^*^	1.94 (1.47, 2.56)^2,*^
No anemia	498	131	1.00 (reference)	1.00 (reference)

RR, relative risk; aRR, adjusted relative risk; CI, confidence interval.

1 aRR analysis with Mantel-Haenszel estimated risk stratification by weighted combined maternal pre-pregnancy body mass index, child stunting status, whether the child was still breastfed, and whether the child was given water before 6 months of age.

2 aRR analysis with Mantel-Haenszel estimated risk stratification by area of residence.

† p<0.1,

* p<0.05.

**Table 4:** Risk stratification of potential confounding variables based on maternal pre-pregnancy anemia status

Stratification variables	Maternal pre-pregnancy anemia status	n	Cases of child anemia (n=73)	Incidence rate of child anemia (%)	RR (95% CI)
Child stunting (n=630)					
Yes	Anemia	91	19	20.9	1.64 (1.00, 2.70)^†^
	No anemia	291	37	12.7	1.00 (reference)
No	Anemia	44	5	11.4	2.58 (0.91, 7.31)
	No anemia	204	9	4.4	1.00 (reference)
Child still breastfed (n=613)					
Yes	Anemia	39	14	35.9	2.11 (1.16, 3.86)^*^
	No anemia	100	17	17.0	1.00 (reference)
No	Anemia	92	10	10.9	1.43 (0.72, 2.83)
	No anemia	382	29	7.6	1.00 (reference)
Child fed water early (n=614)					
Yes	Anemia	101	17	16.8	2.08 (1.20, 3.61)^*^
	No anemia	384	31	8.1	1.00 (reference)
No	Anemia	31	8	25.8	1.69 (0.79, 3.60)
	No anemia	98	15	15.3	1.00 (reference)
Maternal pre-pregnancy BMI (n=631)					
Underweight and Normal	Anemia	102	21	20.6	1.94 (1.20, 3.14)^*^
	No anemia	367	39	10.6	1.00 (reference)
Overweight and Obese	Anemia	35	4	11.4	2.07 (0.64, 6.68)
	No anemia	127	7	5.5	1.00 (reference)
Maternal anemia in 2014 (n=636)					
Yes	Anemia	68	12	17.6	0.80 (0.44, 1.46)
	No anemia	131	29	22.1	1.00 (reference)
No	Anemia	70	13	18.6	2.20 (1.21, 3.99)^*^
	No anemia	367	31	8.4	1.00 (reference)

BMI, body mass index; RR, relative risk; CI, confidence interval.

† p<0.1,

* p<0.05.

## References

[1] Wang L, Li M, Dill SE, Hu Y, Rozelle S (2019). Dynamic anemia status from infancy to preschool-age: evidence from rural China. Int J Environ Res Public Health.

[2] Dos Santos RF, Gonzalez ES, de Albuquerque EC, de Arruda IK, Diniz Ada S, Figueroa JN (2011). Prevalence of anemia in under five-year-old children in a children’s hospital in Recife, Brazil. Rev Bras Hematol Hemoter.

[3] Stevens GA, Finucane MM, De-Regil LM, Paciorek CJ, Flaxman SR, Branca F (2013). Global, regional, and national trends in haemoglobin concentration and prevalence of total and severe anaemia in children and pregnant and non-pregnant women for 1995–2011: a systematic analysis of population-representative data. Lancet Glob Health.

[4] Geller SE, Koch AR, Garland CE, MacDonald EJ, Storey F, Lawton B (2018). A global view of severe maternal morbidity: moving beyond maternal mortality. Reprod Health.

[5] United Nation Children’s Fund (UNICEF) (2018). Nutrition capacity assessment in Indonesia.

[6] Ministry of Health Republic of Indonesia (2021). Iron folic acid tablet supplementation guidelines.

[7] Malako BG, Asamoah BO, Tadesse M, Hussen R, Gebre MT (2019). Stunting and anemia among children 6–23 months old in Damot Sore district, Southern Ethiopia. BMC Nutr.

[8] Balarajan Y, Ramakrishnan U, Ozaltin E, Shankar AH, Subramanian SV (2011). Anaemia in low-income and middle-income countries. Lancet.

[9] Ministry of Health Republic of Indonesia (2013). Basic health research.

[10] Agency for Health Research and Development (2019). Indonesia basic health research (RISKESDAS) 2018.

[11] World Health Organization Maternal, newborn, child and adolescent health and ageing: data portal.

[12] Onyeneho NG, Ozumba BC, Subramanian SV (2019). Determinants of childhood anemia in India. Sci Rep.

[13] Ezzati M, Lopez AD, Rodgers AA, Murray CJ (2004). Comparative quantification of health risks: global and regional burden of disease attributable to selected major risk factors.

[14] Garzon S, Cacciato PM, Certelli C, Salvaggio C, Magliarditi M, Rizzo G (2020). Iron deficiency anemia in pregnancy: novel approaches for an old problem. Oman Med J.

[15] Nguyen PH, Young M, Gonzalez-Casanova I, Pham HQ, Nguyen H, Truong TV (2016). Impact of preconception micronutrient supplementation on anemia and iron status during pregnancy and postpartum: a randomized controlled trial in rural Vietnam. PLoS One.

[16] Ntenda PA, Nkoka O, Bass P, Senghore T (2018). Maternal anemia is a potential risk factor for anemia in children aged 6–59 months in Southern Africa: a multilevel analysis. BMC Public Health.

[17] Allen LH (2000). Anemia and iron deficiency: effects on pregnancy outcome. Am J Clin Nutr.

[18] Atrash HK, Johnson K, Adams M, Cordero JF, Howse J (2006). Preconception care for improving perinatal outcomes: the time to act. Matern Child Health J.

[19] Strauss J, Witoelar F, Sikoki B, Wattie AM (2009). The fourth wave of the Indonesia Family Life Survey (IFLS 4): overview and field report.

[20] Strauss J, Witoelar F, Sikoki B (2016). The fifth wave of the Indonesia Family Life Survey: overview and field report.

[21] Mason E, Chandra-Mouli V, Baltag V, Christiansen C, Lassi ZS, Bhutta ZA (2014). Preconception care: advancing from ‘important to do and can be done’ to ‘is being done and is making a difference’. Reprod Health.

[22] Ministry of Health Republic of Indonesia (2008). Basic health research 2007.

[23] World Health Organization Nutrition landscape information system (NLiS): low birth weight.

[24] World Health Organization (2017). Guideline: protecting, promoting and supporting breastfeeding in facilities providing maternity and newborn services.

[25] Kothari MT, Coile A, Huestis A, Pullum T, Garrett D, Engmann C (2019). Exploring associations between water, sanitation, and anemia through 47 nationally representative demographic and health surveys. Ann N Y Acad Sci.

[26] Adu P, Attivor W, Nartey ST, Ephraim RK, Awuku YA (2020). Low iron stores in preconception nulliparous women; a two-center cross-sectional study in peri-urban Ghana. Nutrition.

[27] Ronnenberg AG, Wood RJ, Wang X, Xing H, Chen C, Chen D (2004). Preconception hemoglobin and ferritin concentrations are associated with pregnancy outcome in a prospective cohort of Chinese women. J Nutr.

[28] Abu-Ouf NM, Jan MM (2015). The impact of maternal iron deficiency and iron deficiency anemia on child’s health. Saudi Med J.

[29] Coates TD (2014). Physiology and pathophysiology of iron in hemoglobin-associated diseases. Free Radic Biol Med.

[30] Semba RD, Bloem MW (2002). The anemia of vitamin A deficiency: epidemiology and pathogenesis. Eur J Clin Nutr.

[31] Yin S, Zhou Y, Li H, Cheng Z, Zhang Y, Zhang L (2020). Association of maternal BMI during early pregnancy with infant anemia: a large Chinese birth cohort. Nutr Metab (Lond).

[32] Yang W, Li X, Li Y, Zhang S, Liu L, Wang X (2012). Anemia, malnutrition and their correlations with socio-demographic characteristics and feeding practices among infants aged 0–18 months in rural areas of Shaanxi province in northwestern China: a cross-sectional study. BMC Public Health.

[33] Gaston RT, Habyarimana F, Ramroop S (2022). Joint modelling of anaemia and stunting in children less than five years of age in Lesotho: a cross-sectional case study. BMC Public Health.

[34] Agustina R, Wirawan F, Sadariskar AA, Setianingsing AA, Nadiya K, Prafiantini E (2021). Associations of knowledge, attitude, and practices toward anemia with anemia prevalence and height-for-age z-score among Indonesian adolescent girls. Food Nutr Bull.

[35] Mohammed SH, Larijani B, Esmaillzadeh A (2019). Concurrent anemia and stunting in young children: prevalence, dietary and non-dietary associated factors. Nutr J.

[36] De Sanctis V, Soliman A, Alaaraj N, Ahmed S, Alyafei F, Hamed N (2021). Early and long-term consequences of nutritional stunting: from childhood to adulthood. Acta Biomed.

[37] Meinzen-Derr JK, Guerrero ML, Altaye M, Ortega-Gallegos H, Ruiz-Palacios GM, Morrow AL (2006). Risk of infant anemia is associated with exclusive breast-feeding and maternal anemia in a Mexican cohort. J Nutr.

[38] Marques RF, Taddei JA, Lopez FA, Braga JA (2014). Breastfeeding exclusively and iron deficiency anemia during the first 6 months of age. Rev Assoc Med Bras (1992).

