# Supplementary material for: Maternal pre-pregnancy anemia and childhood anemia in Indonesia: a risk assessment using a population-based prospective longitudinal study
Source: Epidemiol Health. 2022 Nov 1;44:e2022100. doi: 10.4178/epih.e2022100 (PMC10106539; doi:10.4178/epih.e2022100)
Supplement: Supplementary file 1 [file epih-44-e2022100-Supplementary-1.docx]

| **Supplementary Material 1. Distribution of selected and unselected baseline population (n=990)** | | | | | | | | |  |  |  |  |
| --- | --- | --- | --- | --- | --- | --- | --- | --- | --- | --- | --- | --- |
| **Variables** |  | | **Pre-pregnant women in the baseline** | | |  |  |  |  |  |  |  |
|  | **N** | | **Selected** | | **Not selected** | **p-value^a^** |  |  |  |  |  |  |
|  |  | | **(n=616)** | | **(n=374)** |  |  |  |  |  |  |  |
| Age in 2007^b^ | 990 | | 27.10 ± 5.24 | | 26.10 ± 4.99 | 0.00* |  |  |  |  |  |  |
| Pre-pregnancy iron supplement consumption (n=951) | | | | | | | | |  |  |  |  |
| Consumed | 815 | | 512 | | 303 | 0.69 |  |  |  |  |  |  |
| Not consumed | 136 | | 83 | | 53 |  |  |  |  |  |  |  |
| Number of iron supplement tablet consumed in the last 4 weeks (2007)^b^ | 723 | | 111.66 ± 123.48 | | 119.79 ± 123.17 | 0.39 |  |  |  |  |  |  |
| Pre-pregnancy BMI (n=982) | | | | | | | | |  |  |  |  |
| Underweight | 96 | | 60 | | 36 | 0.42 |  |  |  |  |  |  |
| Normal | 645 | | 394 | | 251 |  |  |  |  |  |  |  |
| Overweight | 197 | | 130 | | 67 |  |  |  |  |  |  |  |
| Obese | 44 | | 26 | | 18 |  |  |  |  |  |  |  |
| Smoking during pre-pregnancy (n=988) | | | | | | | | |  |  |  |  |
| Yes | 7 | | 5 | | 2 | 0.62 |  |  |  |  |  |  |
| No | 981 | | 610 | | 371 |  |  |  |  |  |  |  |
| Living area in 2007 (n=518) | | | | | | | | |  |  |  |  |
| Urban | 258 | | 175 | | 83 | 0.69 |  |  |  |  |  |  |
| Rural | 284 | | 172 | | 88 |  |  |  |  |  |  |  |
| Region in Indonesia (n=890) | | | | | | | | |  |  |  |  |
| Sumatera | 184 | | 117 | | 67 | 0.29 |  |  |  |  |  |  |
| Java and Bali | 452 | | 273 | | 179 |  |  |  |  |  |  |  |
| Other provinces | 172 | | 119 | | 53 |  |  |  |  |  |  |  |
| Mother’s education in 2007 (n=972) | | | | | | | | |  |  |  |  |
| Elementary or equal | 304 | | 201 | | 103 | 0.02* |  |  |  |  |  |  |
| Junior high or equal | 244 | | 161 | | 83 |  |  |  |  |  |  |  |
| Senior high or equal | 307 | | 177 | | 130 |  |  |  |  |  |  |  |
| Undergraduate and graduate | 117 | | 67 | | 50 |  |  |  |  |  |  |  |
| Maternal pre-pregnancy anemia (n=990) | |  | |  | | | | | |  |  |  |
| Anemia | 219 | | 133 | | 86 | 0.61 |  |  |  |  |  |  |
| No anemia | 771 | | 483 | | 288 |  |  |  |  |  |  |  |
| ^a^ p<0.05 considered significant; analysis using t-test for linear variable and ANOVA for categorical variable | | | | | | | | |  |  |  |  |
| ^b^ presented in Mean ± SD | | | | | | | | |  |  |  |  |
